# Development of Mechanical Properties of Stainless Steel 316LN-IG after Cryo-Plastic Deformation

**DOI:** 10.3390/ma16196473

**Published:** 2023-09-29

**Authors:** Alica Fedoriková, Patrik Petroušek, Tibor Kvačkaj, Róbert Kočiško, Michal Zemko

**Affiliations:** 1 Department of Material Analysis, Research Centre Řež Ltd., Hlavní 130, 250 68 Husinec, Czech Republic; alica.fedorikova@cvrez.cz; 2 Department of Plastic Deformation and Simulation Processes, Institute of Materials and Quality Engineering, Faculty of Materials, Metallurgy and Recycling, Technical University of Kosice, Park Komenského 11, 040 01 Kosice, Slovakia; robert.kocisko@tuke.sk; 3 Bodva Industry and Innovation Cluster, Budulov 174, 045 01 Moldava nad Bodvou, Slovakia; tibor.kvackaj@tuke.sk; 4 COMTES FHT a.s., Průmyslová 995, 334 41 Dobřany, Czech Republic; michal.zemko@comtesfht.cz

**Keywords:** mechanical properties, 316LN-IG stainless steel, cryogenic treatment, rolling, heat treatment

## Abstract

The article deals with increasing the mechanical properties of stainless steel 316 Ln-IG, which is intended for work in cryogenic temperatures (liquid nitrogen and liquid helium), such as conductor conduits for the ITER magnet system. The strength and plastic properties were increased by a combination of cold and cryo-rolling and heat treatment. The mechanical properties of rolled material were investigated at 293 K, 77 K, and 4.2 K. The work-hardening rate of the steel increased continuously with a lowering of the temperature. The maximum yield strength and ultimate tensile strength were achieved by the cryo-rolling process with a total thickness deformation of 50%. The material properties tested at ambient temperature were 0.2YS = 1050 MPa, UTS = 1200 MPa, and at 4.2 K, the values were 0.2YS = 1804 MPa and UTS = 2081 MPa. Two types of long-term heat treatment were applied after experimental rolling (823 K and 1093 K for 10 h). The highest precipitation hardening of steel was achieved at a temperature of 823 K after 50% deformation. The resulting grain size decreased from the initial 216 μm (before the rolling process) to 70 μm after ambient rolling and 72 μm after cryo-rolling.

## 1. Introduction

Due to its good corrosion resistance and malleability, austenitic stainless steel is one of the most attractive construction materials in the energy industry [1,2,3]. Therefore, the development of steels is aimed at stainless steels and their improvement, due to their wide use in cryogenic temperatures as a structural material in fusion reactors to the red-hot temperatures of furnaces and engines [4,5,6,7]. For devices operating at liquid helium temperatures, such as conductor conduits for the International Thermonuclear Experimental Reactor (ITER) magnet system, 316LN-IG stainless steel is used [8]. The presence of chromium secures the excellent corrosion resistance of these iron-based alloys at a minimum of about 12 wt.%. Some other alloying elements are also used to improve the properties of stainless steel, e.g., nickel, molybdenum, titanium, manganese, silicon, niobium, selenium, and nitrogen. All stainless steels have carbon, normally present in an amount ranging from less than 0.03% to 0.1 wt.% in certain grades [9]. Another important alloying element is nitrogen, which has a stabilizing effect on austenite and also exhibits a strong interstitial strengthening effect [10,11]. The properties of austenitic stainless steels can be influenced by thermo-mechanically controlled processing treatments, and the strength of an originally soft material (0.2YS~200 MPa) can be improved up to 1600 MPa. Improvement of the mechanical properties of austenitic stainless steels is possible by severe plastic deformation methods [3,12]. The low temperature of deformation would prevent dynamic recovery and stimulate mechanical twinning, which enhances the grain refinement effect [3,13,14]. Subsequently, the deformation twins continued to coordinate the severe plastic deformation. At low strain, the mechanism of plastic deformation is dislocation propagation and slip, while at higher strain the main mechanism becomes mechanical twinning [14]. It can be assumed that, with further increasing strain, the refinement of grain size by the development of high-density mechanical twins occurs [15]. Thus, deformation at low temperatures is the typical formation of high-density twins, representing many twin boundaries, and the origin grains are divided into twin-matrix lamellae. With increasing strain, when the formation of mechanical twins becomes energy-intensive and difficult without the thin lamellae inside the divided grains, the dislocation activities become functional and release further plastic deformation. Dislocations in the twin-matrix lamellae organize themselves into dislocation walls to minimize the strain energy. During cold forming, a phenomenon of second-order twinning was investigated. The formation of second-order twins (SOT), or secondary twins, also takes place within the mother twin (MT) in an already twinned section of the crystal [16]. It may involve analogous geometrical degrees of freedom and occur due to the interaction of perfect glide and twinning or due to the intersection of twins [17,18].

The first information about the rolling of stainless steels under cryogenic conditions (CR) was published by Üçok [19] in 1991. It presented the wider applications of CR techniques for increasing yield strength in stainless steels [12,20,21,22,23].

The plastic deformation of stainless steels carried out at cryogenic temperatures leads to a remarkable increase in strength and hardness, but also to a decrease in elongation. The studies [24,25] have shown that with the final nanograined structure the size level can obtain a yield strength up to 0.2YS ≈ 2000 MPa, which is very attractive for technical applications. The mechanisms leading to the rise in the strength properties can be defined as follows: solid solution strengthening, strengthening from grain-size refinement, strain strengthening, precipitation strengthening, and transformation strengthening. 

In stainless steel, different types of precipitates may form during heat treatment or thermal aging. As main products of precipitation in 316LN-IG stainless steel, carbides M_23_C_6_ and M_6_C and intermetallic phases such as phase χ (Chi), σ (Sigma), and η (Laves) may be considered [26,27,28]. A significant number of carbides can be formed after only annealing for a few minutes at a temperature interval from 650 to 750 °C [29]. With a strongly deformed structure, softening of the structure as annihilation of dislocations and rearrangement of grain boundaries will also take place during these thermally active events. The interaction of these events can influence the mechanical properties of stainless steel very significantly. 

This study presents the development of the mechanical properties of 316LN-IG stainless steel after cryo-plastic deformation and subsequent heat treatment. The influence of plastic deformations during rolling at ambient temperature and in cryogenic conditions was compared. To evaluate the mechanical properties, a static tensile test was carried out at temperatures of 4.2 K, 77 K, and ambient temperature. Structural analysis of different deformation states with subsequent heat treatment was carried out using optical microscopy.

## 2. Materials and Methods

### 2.1. Experimental Material

For the experiment, austenitic stainless steel of grade 316LN-IG was used (C-0.06 wt.%; Cr-18.76 wt.%; Ni-13.73 wt.%; Mn-1.5 wt.%; Mo-1.87 wt.%; N-0.13 wt.%; and Fe-Bal.). The material was produced by COMTES FHT a.s. (Dobřany, Czech Republic). The chemical composition was determined using an emission spectrometer, a Bruker Q4 TASMAN 130 (Bruker Elemental GmbH, Kalkar, Germany).

After melting and casting, the hot forging of the ingot was performed. The forging temperature range was $T=\left\langle 1098-\left.1473\right\rangle \right.\;\left[K\right]$. From the middle part of a hot-forged ingot, the rectangular samples for experimental rolling were cut. The input dimension of the material for rolling was $h_{0}\times b_{0}\times l_{0}=15\times 40\times 75\;mm$. These were subsequently treated by solid solution annealing (SA) in the atmospheric furnace at 1323 K for 1 h, with fast cooling to ambient temperature.

### 2.2. Physical Simulations—Experimental Rolling

Two types of experimental unidirectional rolling were carried out: conventional rolling at ambient temperature (AR) and rolling at cryogenic temperature (CR). Samples were rolled with about 10% reduction for each pass, and the total thickness deformation was $\varepsilon =\left\langle 10;30;\left.50\right\rangle \right.\;\left[\%\right]$. The cryogenic rolling conditions were secured by immersing the specimens in liquid nitrogen (77 K) for 0.5 h before the rolling process and for 10 min immediately after each pass. In the case of cryogenic rolling, the temperature of samples after passing the rolling mill was in the range T_CT_ = <153; 183> [K]. The experimental rolling process was carried out in a four-high roll arrangement (COMTES FHT a.s., Czech Republic).

### 2.3. Differential Scanning Calorimetry (DSC)

Samples for DSC analysis were cut using the wire-cutting machine. The input weight of the samples was 20 mg. The DSC analysis was performed with a heating rate of 30 K/min in a nitrogen atmosphere using the simultaneous thermal analyzer, a STA 449 F1 Jupiter (Netzsch, Waldkraiburg, Germany).

### 2.4. Heat Treatment

After rolling, heat treatments $T_{HT}=\langle 473;773;873;973;1073\rangle \;\left[K\right]$ in the atmospheric furnace were selected, after which a hardness test was carried out to determine suitable temperatures for further processing. Holding time was 0.5 h. The annealing was followed by rapid cooling in water. Annealed material for hardness measurement was used. Based on the hardness measurement and the DSC analysis, the heat treatments shown in Table 1 were chosen for further processing. The annealing process in the atmospheric furnace was followed by rapid cooling in water to fix the structure.

As received material after solution annealing, it has the label SA. The system of sample labeling after rolling and heat treatment is shown in Table 2.

### 2.5. Mechanical Properties

The static tensile test was carried out at TINIUS OLSEN H300KU (Tinius Olsen, Salfords, England) for ambient temperature and 77 K testing, and at MTS 100 Landmark (MTS, Eden Prairie, MN, USA), which was equipped with a cryostat and an extensometer. 

**Figure 1:**
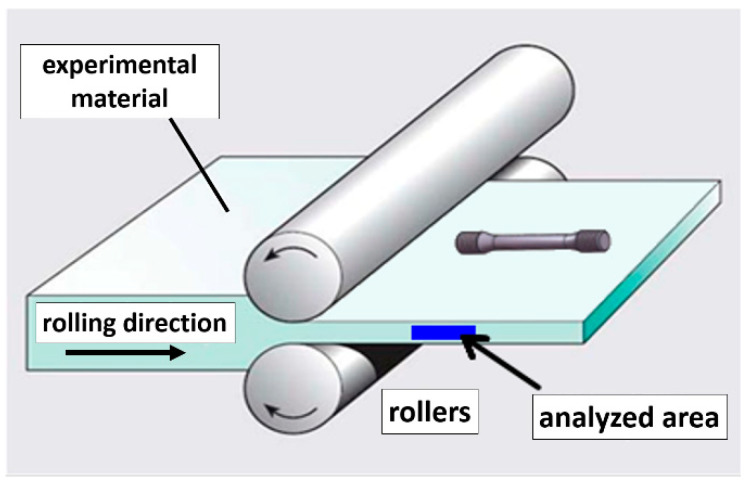
Schematic diagram of the rolling process and analyzed area.

The samples for the static tensile test were cut from material in the longitudinal direction of the rolling process, as shown in Figure 1. For each tensile test, the speed of the crossbar movement was 0.3 mm·min^−1^, which corresponds to a deformation speed of 0.00025 s^−1^. These values correspond to the standard for tensile testing [30]. The initial gear length of the sample for the static tensile test was L_0_ = 22 mm. At the ambient temperature and at 77 K for the static tensile tests, at least three tests were carried out for all deformed states and heat treatments. At a temperature of 4.2 K, there were two tests for each state.

The Vickers hardness test HV30 was carried out using the hardness tester HPO 250. The total load for testing was 294 N.

### 2.6. Microstructure

Cross-sectional samples for light optical microscopy (LOM) analysis were cut from rolled material parallel to the rolling direction with a saw with a water cooling system using Accutom 100 (Struers, Ballerup, Denmark). The samples were ground and polished by standard procedures recommended by Struers, on the Tegramin 25 machine (Struers, Ballerup, Denmark). Polished samples were etched in two different etchants: aquaregia solution, which contained hydrochloric acid and nitric acid in a volume ratio of 1:3 (HCl: HNO_3_ = 3:1), and methanolic aquaregia diluted with methanol in a volume ratio of HCl: HNO_3_: methanol = 3:1:1. The etching time varied from a few seconds to minutes, depending on total deformation and heat treatment conditions.

The samples were examined with a Leica DM 2700M optical microscope (Leica Microsystems, Wetzlar, Germany). Digital micrographs of 2560 × 1920 pixels were taken for each sample. The magnifications of 50×, 200×, and 500× were used. The micrographs were analyzed according to the standard for determining grain size [31]. 

## 3. Results and Discussion

### 3.1. Determination of Heat Treatment Using the DSC Analysis

**Figure 2:**
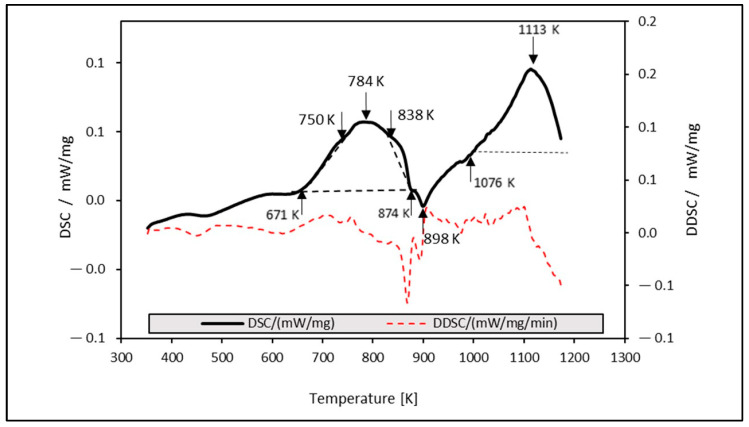
Dependence of the DSC analysis at the temperature used for short-term annealing.

The dependence of heat released for the stainless steel sample after solution annealing versus temperature during constant heating at a rate of 30 K/min is shown in Figure 2. In addition to recovery and recrystallization processes, phase transformations such as precipitation of carbides and nitrides, intermetallic phases χ, σ, and η and reverse martensitic transformations must also be taken into account in austenitic stainless steels [6]. Two large exothermic thermal effects on the *DSC* curve are visible. The first peak is in the temperature range from 671 K to 874 K, which reveals multiple effects. In total, three peaks can be observed, with the main maximum at 784 K and two additional low maxima at about 750 K and 838 K. All these exothermic peaks can be caused by the precipitation of M_23_C_6_ type carbides. The point of formation of M_23_C_6_ (chromium-rich carbide) and intermetallic phases was mentioned in [32,33] at a temperature higher than 823 K. In the paper [34], using the DSC and electrical resistance measurements, the authors identified four distinct stages of precipitation in 316L stainless steel: coherent precipitation, its coarsening and initiation of grain boundary precipitation, σ phase, and, finally, M_23_C_6_ precipitation. From the DSC curve, it is not possible to clearly attribute the temperatures to individual stages of precipitation. The development of precipitates can also be influenced by thermomechanical processing, while large plastic deformation significantly accelerates precipitation [32]. The second exothermic peak with a peak of 1113 K probably represents the initial stage of recrystallization, which is also accompanied by the gradual dissolution of M_23_C_6_ precipitates. This effect was already observed at a temperature of 1123 K [6], and according to the authors, complete recrystallization occurs at a temperature higher than 1323 K. Between the two exothermic reactions, the DSC curve also shows a small endothermic effect in the temperature interval from 673 K to 1023 K with a peak at 898 K, which, according to the study [35], represents a reverse martensitic transformation (α′-martensite to γ-austenite).

To create an optimal synergy between the structural and mechanical properties of structural stainless steel processed using plastic deformations, it is necessary to apply a suitable heat treatment. This must be done in such a way as to reconcile the mutual interaction between softening and precipitation hardening, possibly by recrystallization.

Based on the hardness and the DSC measurements, two annealing temperatures for the steel processing for long-term annealing were determined: HT1 = 823 K and HT2 = 1093 K. The maximum increase in mechanical properties was expected at HT1. The heat treatment at HT2 was used to check the thermal stability of precipitation strengthening. To assess the mechanical properties of the fully recrystallized structure, the thermal treatment HT3 = 1323 K was determined and applied to the samples with the highest deformation (A50 and C50).

### 3.2. Mechanical Properties after Plastic Deformation

#### 3.2.1. Static Tensile Test

**Figure 3:**
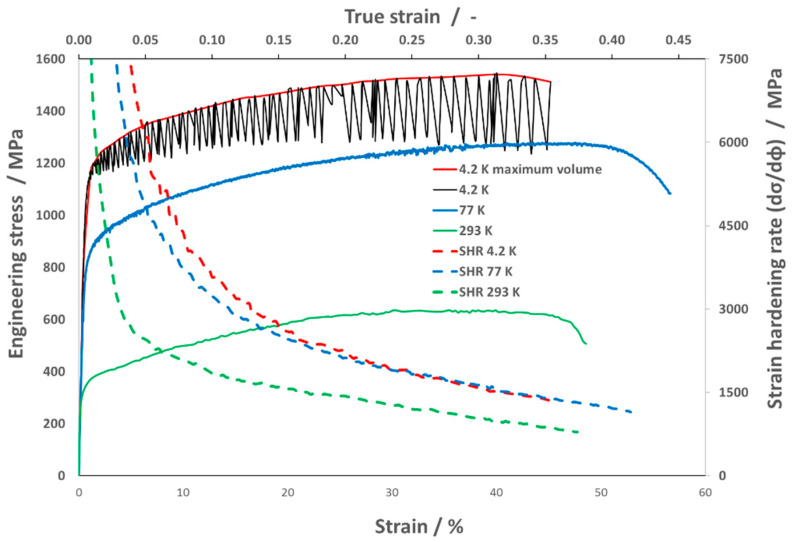
Engineering stress–strain curves with strain hardening rate (SHR) curves for 316 LN-IG stainless steel after SA at liquid helium temperature (4.2 K), at liquid nitrogen (77 K) and at ambient temperature (293 K).

Figure 3 shows the representative graphic dependency of engineering stress—the strain and strain hardening rate—and the true strain at various test temperatures after SA.

The initial state of 316LN-IG after solution annealing has more than 100 MPa higher mechanical properties at ambient temperature than is required for ITER applications with yield strength 0.2YS_SA,293K_ = 325 MPa, ultimate tensile strength UTS_SA,293K_ = 641 MPa and total elongation TE_SA,293K_ = 49%. The ITER applications require a yield strength 0.2YS_SA,293K_ = 220 MPa, ultimate tensile strength UTS_SA,293K_ = 525 MPa, and total elongation TE_SA,293K_ = 45% [6]. 

At ambient temperature, the material shows a high supply of plasticity, where uniform elongation reaches 40% (UTS/0.2YS ≈ 2). At the same time, the most significant strain hardening rate is observed only up to deformation φ = 0.05, after which it gradually decreases.

At liquid nitrogen temperature, the material properties increased significantly, more than twice, where yield strength was 0.2YS_SA,77K_ = 790 MPa and ultimate tensile strength UTS_SA,77K_ = 1282 MPa, and total elongation increased to TE_SA,77K_ = 57%. It is also possible to observe not only an increase in the uniform elongation UE_SA,77K_ = 50%, but also the strain hardening rate, especially up to the value φ = 0.15. These results confirm that the material ASS 316LN-IG, due to its fcc (face-centered cubic) structure, exhibits excellent deformability at cryogenic temperatures.

In the conditions of liquid helium, stress oscillations occur during a single-axis tensile test controlled by displacement, which creates the so-called discontinuous plastic flow (DPF). This phenomenon occurs when the temperature approaches absolute zero, below temperature T1, which for 316L is around 35 K [36]. Two basic hypotheses are known to explain this phenomenon. The first one is based on dislocation dynamics, and the second one is related to thermal effects [37]. The author of [38] would confirm that the DPF has a mechanical origin and is accompanied by thermal effects, based on experimental measurements.

**Figure 4:**
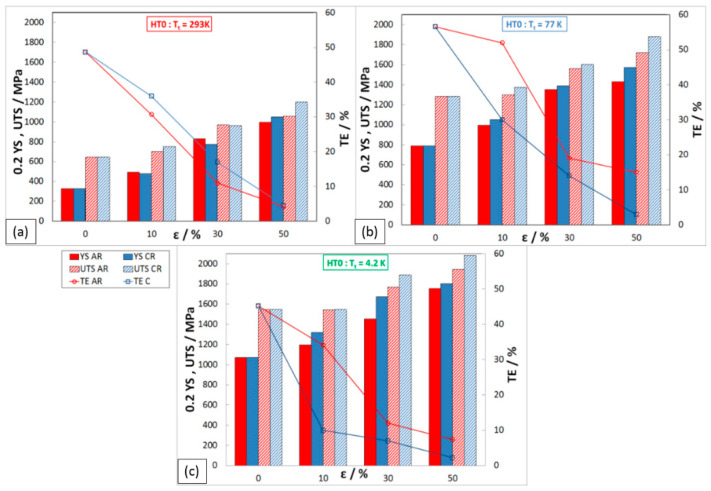
Mechanical properties of rolled material HT0 at different T_t_: (**a**) 293 K, (**b**) 77 K, (**c**) 4.2 K.

The mechanical properties and strain hardening rate of the steel 316 LN-IG tested at 4.2 K were evaluated from the maximum values of plastic flow, which are shown in Figure 4, marked with a red curve. At this temperature, the highest mechanical properties were achieved, and increased almost threefold compared to the properties at ambient temperature: yield strength 0.2YS_SA,4.2K_ = 1070 MPa and ultimate tensile strength UTS_SA,4.2K_ = 1543 MPa, while the total elongation was maintained at the same value, TE_SA,4.2K_ = 45%. The plastic flow in the initial stage shows a further increase in the strain hardening rate.

The results of the static tensile test of rolled material without any annealing process (HT0) are presented in Figure 4. All three temperatures of the test are shown: (a) 293 K; (b) 77 K; and (c) 4.2 K. The red color of the column presents the ambient rolling process and the blue the cryogenic rolling process. Yield strength (0.2YS) is shown by the full-colored bar, ultimate tensile strength (UTS) by the shaded bar, and total elongation (TE) is shown by a line graph on the secondary y-axis. 

After the individual deformations in rolling, there is a significant strengthening of the material but also a dramatic reduction in plasticity when the total elongation, in some cases, reaches the value of TE_C50%,4.2K_ = 2.2%.

The maximal level of strength properties of material processing by ambient rolling with 50% total deformation was obtained: 0.2YS_A50,293K_ = 994 MPa, UTS_A50,293K_ = 1055 MPa, with a minimum of TE_A50,293K_ = 4.1%. Higher strength properties were achieved when the material was processed by cryo-rolling, with 50% of total deformation: 0.2YS_C50,293K_ = 1050 MPa; UTS_C50,293K_ = 1200 MPa; and TE_C50,293K_ = 4.6%.

In the tensile test at ambient temperature, the material shows a higher 0.2YS after 10% and 30% deformations when rolling at cryogenic temperature. In comparison, after 50% deformations, the observed trend is the opposite. The lower 0.2YS at cryo-rolling (10% and 30%) is probably due to the mixed mechanism of plastic deformation (slip and twinning), which can lead to heterogeneity in the strengthening of the structure. After testing the material in cryogenic conditions (77 K and 4.2 K), it is observed that higher strengthening values (0.2YS and UTS) are achieved after rolling at cryogenic temperatures than after ambient rolling. This is probably because the low testing temperatures promote plastic deformation by twinning.

The maximal level of strength properties at the testing temperature of 77 K was obtained by cryo-rolling with 50% deformation: 0.2YS_C50,77K_ = 1571 MPa; UTS_C50,77K_ = 1880 MPa; and a minimum of TE_C50,77K_ = 3%. Mechanical properties (0.2YS, UTS) after ambient rolling with 50% deformation showed 9% lower values than after rolling in cryogenics.

**Figure 5:**
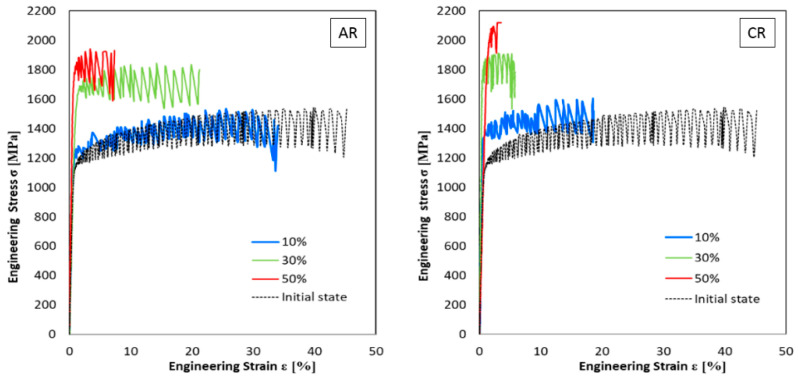
Engineering stress–strain curves with different AR and CR and different deformations at liquid helium temperature (4.2 K).

The curves from static tensile tests carried out at a temperature of 4.2 K on the samples after rolling at ambient and cryogenic temperatures are given in Figure 5. The flow stress of all deformed states tested at a temperature of 4.2 K has the character of discontinuous plastic flow. At this testing temperature, the highest values of the strength properties of the material were measured. The maximal level of strength properties was obtained for ambient rolling samples with 50% deformation and tested at 4.2 K: 0.2Y_SA50,4.2K_ = 1755 MPa, UTS_A50,4.2K_ = 1940 MPa, and a minimum of TE_A50,4.2K_ = 7%. The higher strength properties were achieved when the material was processed by cryo-rolling at 4.2 K: 0.2YS_C50,4.2K_ = 1804 MPa, UTS_C50,4.2K_ = 2081 MPa, and a minimum of TE_C50,4.2K_ = 2%.

#### 3.2.2. Structure Development after Plastic Deformations

**Figure 6:**
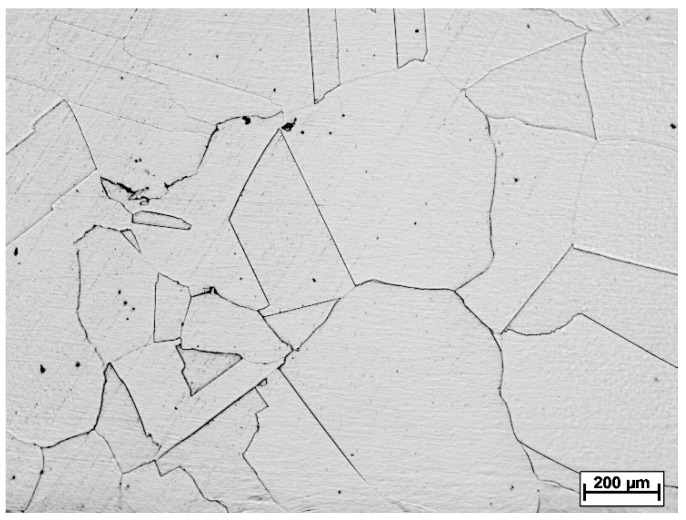
Experimental material before the rolling process—solution annealed (SA).

The initial state structure of 316LN austenitic stainless steel (referred to below as SA) is shown in Figure 6. The microstructure can be described as a coarse-grained polyhedric austenite with a small occurrence of annealing twins. The grain size of 316LN stainless steel after solution annealing and before the rolling process was realized according to standard [31]. After the solution annealing, grains were characterized by G numbers. The average diameter of grains was 213.6 μm and G = 1.5.

**Figure 7:**
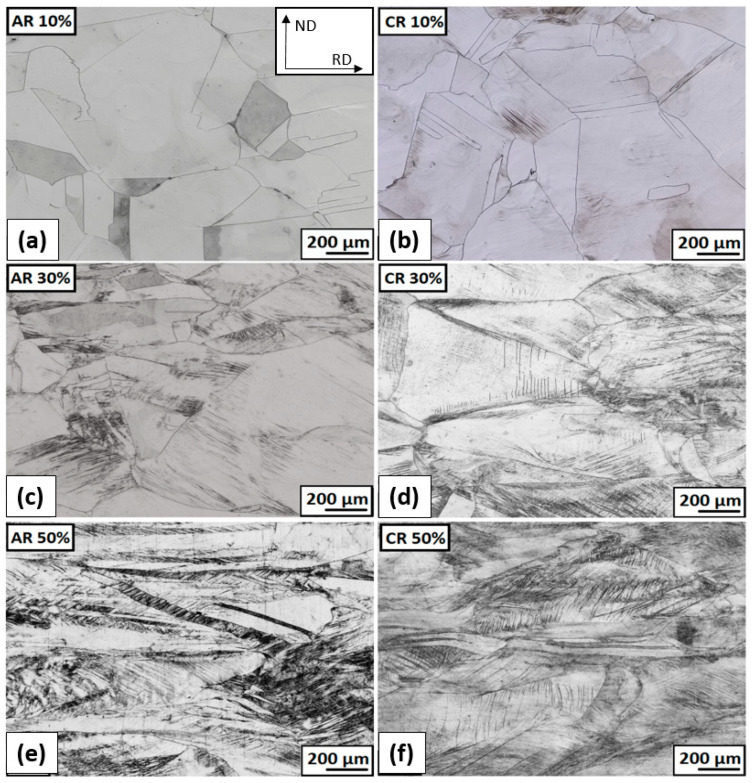
The microstructure overview after ambient and cryogenic rolling with different total thickness deformations without following heat treatment: (**a**) AR; ε = 10% (**b**) CR; ε = 10% (**c**) AR; ε = 30% (**d**) CR; ε = 30% (**e**) AR; ε = 50% (**f**) CR; ε = 50%.

The micrographs of the deformed structure after ambient and cryogenic rolling (without heat treatment) are shown in Figure 7. The 10% deformation at ambient temperature rolling (AR 10%) causes former grain boundaries, annealing twins, and grains with original grain sizes that are still visible. An inhomogeneous gray contrast within single grains results from an increasing number of dislocations. Former grain boundaries, a few annealing twins, and grains with original grain sizes are still visible. After 10% of deformations in cryogenic conditions, a hint of the first deformation bands, which are generated from the original grain boundaries, can be seen in some grains.

**Figure 8:**
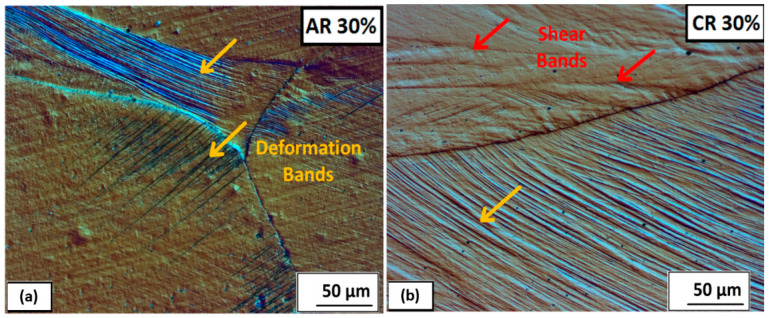
Deformation bands and shear bands visible in austenite grains after 30% deformation: (**a**) ambient rolling, (**b**) cryogenic rolling.

A significant number of deformation bands are formed in some grains, even at a strain of 30% thickness deformation. The detailed deformation bands and shear bands are shown in Figure 8 for ambient and cryogenic rolling after ε = 30%. The differential interference contrast filter (DIC) was used to increase the contrast and spatial display of the microstructure. On the microstructure, it is visible that deformation bands are created in separate grains and end at the grain boundary. A higher proportion are in the structure after rolling at cryogenic temperatures, and the first shear bands are also observed. The angle of the bands varies and depends on grain orientation. A few grains are oriented in the rolling direction (A 30%, C 30%). 

After the rolling at ambient temperature and ε = 50% (AR 50%) deformation, former grain boundaries and grains of the original size are slightly visible. A high number of deformation bands and low-angle grain boundaries are observed in all grains. More grains tilt in the rolling direction (a pancake-like shape).

The grain size decreases with increasing levels of total thickness deformation ε. From solution-annealed grains with an average diameter of approx. 200 μm, the grain size has changed to 77 μm after 30% ambient rolling and 64 μm after cryo-rolling, respectively. In the case of 50% total-thickness deformation, an evaluation of grain size is slightly complicated, due to the strong deformation of the microstructure, and grain boundaries are no longer clearly visible. The deformation is inhomogeneous. The grains are elongated in the rolling direction. In the direction of the rolling thickness (ND), the grains have dimensions from 10 to 200 µm. In between these huge, elongated grains, the strongly deformed microstructure, made up of tangles of deformation bands, shear bands, and twins, was observed. The small grains without well-defined boundaries have a diameter of 50 μm or less.

**Figure 9:**
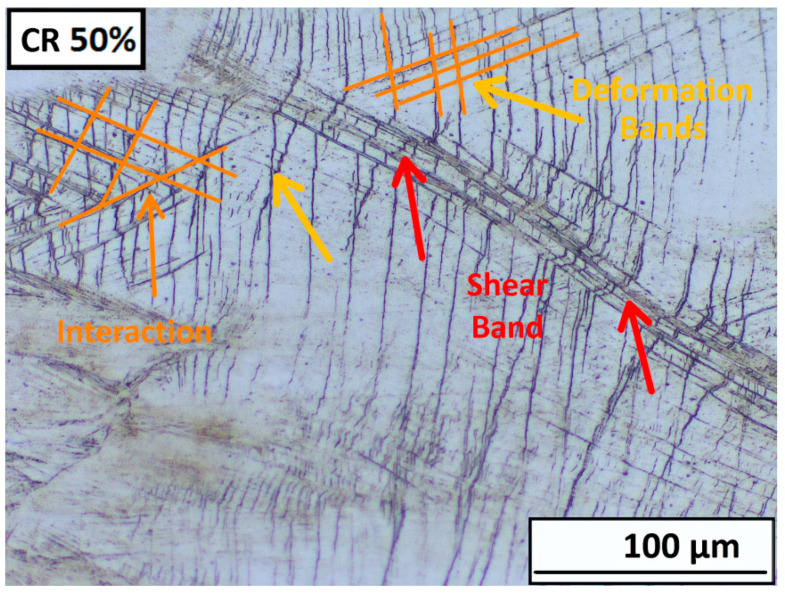
Detail of the mutual interaction of deformation band and share bands in ε = 50% ambient rolled sample.

A detailed description of the mutual interaction between the shear bands and deformation bands is shown in Figure 9. Here, it is clearly seen that the shear bands and deformation bands cross each other and even overcome the boundaries of the original grains. In the case of cryogenic rolling, this process is more significant. The angle of the bands varies and depends on grain orientation.

### 3.3. Properties Stainles Steel 316 LN-IG after Heat Treatment

Heat treatment according to conditions HT1 and HT2 was carried out on samples that were rolled at ambient temperature and in liquid nitrogen with deformations of 10, 30, and 50%. The samples of the HT3 state were deformed by 50% (AR and CR).

#### 3.3.1. Static Tensile Test

**Figure 10:**
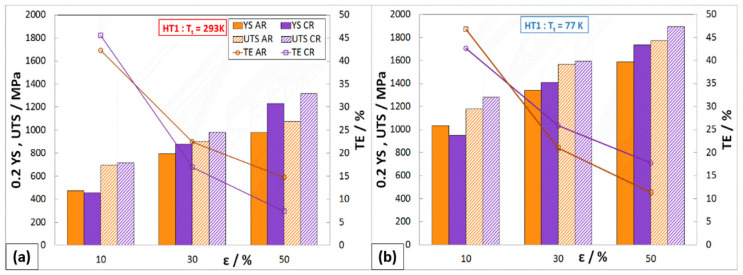
Mechanical properties of rolled material HT1 at different Tt: (**a**) 293 K, (**b**) 77 K.

The development of mechanical properties after HT1 is shown in Figure 10. The strength properties of austenitic steel 316LN-IGS after 10% deformations (AR and CR) show relatively small changes, due to the influence of HT1. In some cases, there was also a decrease in YS and UTS of about 40 MPa compared to the HT0 state. On the other hand, HT1 has a positive effect on ductility, which increased by about 12%. This change in mechanical properties indicates that partial strengthening of the deformed structure may have occurred at the tested temperature. A similar trend in YS, UTS, and TE is also observed on samples after 30% deformations. The beneficial effect of HT1 on the comprehensive improvement of mechanical properties was achieved on samples after 50% deformation rolled in cryogenic conditions, where 0.2YS_C50,293K,HT1_ = 1230 MPa, UTS_C50,293K,HT1_ = 1315 MPa, and TE_C50,293K,HT1_ = 7.4%. This condition when tested in liquid nitrogen also achieved a significant increase in mechanical properties, where 0.2YS_C50,77K,HT1_ = 1735 MPa, UTS_C50,77K,HT1_ = 1895 MPa, and TE_C50,77K,HT1_ = 11.4%.

##### Mechanical Properties after HT2 (1093 K/10 h)

**Figure 11:**
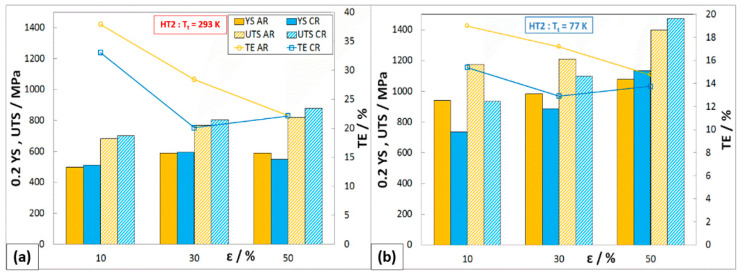
Mechanical properties of rolled material HT2 at different T_t_: (**a**) 293 K, (**b**) 77 K.

The heat treatment HT2 caused a sharp decrease in strength properties, mainly for states after 30% and 50% deformations (AR and CR). All deformation states show very similar strength properties at ambient temperature, where YS ≈ 590 MPa and UTS ≈ 800 MPa (Figure 11). Under these heat treatment conditions, ductility increased to 22%. By testing in cryogenic conditions (77 K), it is possible to see the greatest decrease in strength properties on samples after 50% cryo-deformation: 0.2YS_C50,77K,HT2_ = 1135 MPa, UTS_C50,77K,HT2_ = 1475 MPa, and TE_C50,77K,HT2_ = 14%.

##### Mechanical Properties after HT3 (1323 K/1.5 h)

**Figure 12:**
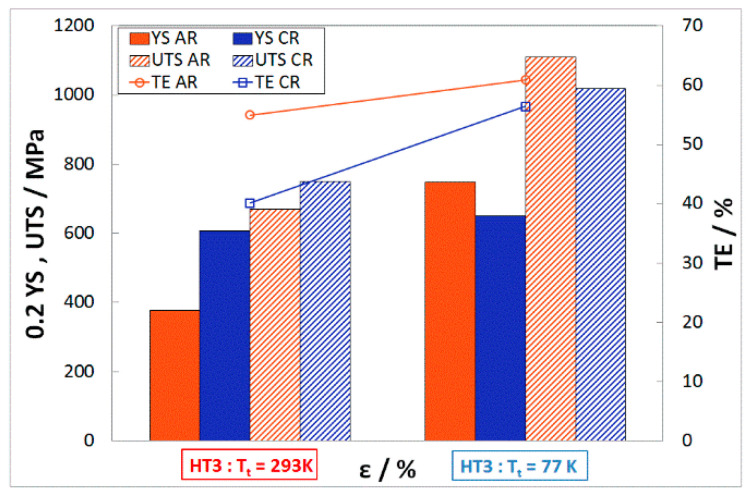
Mechanical properties of A50 and C50—HT3 at different T_t_.

The heat treatment HT3 was carried out to determine the mechanical properties in cases where the temperature of the material reaches the recrystallization temperature. The mechanical properties of stainless steel 316LN-IG after 50% deformation (AR, CR) followed by solid solution annealing (HT3) are shown in Figure 12. The strength properties after HT3 tested at ambient temperature for AR drop to the level of the properties of the base material0.2YS_A50,77K,HT3_ = 377 MPa, UTS_A50,77K,HT3_ = 669 MPa, and TE_A50,77K,HT3_ = 55%, while these values are noticeably higher after CR. Even when tested in cryogenic conditions, the material shows lower strength properties than the base material.

**Figure 13:**
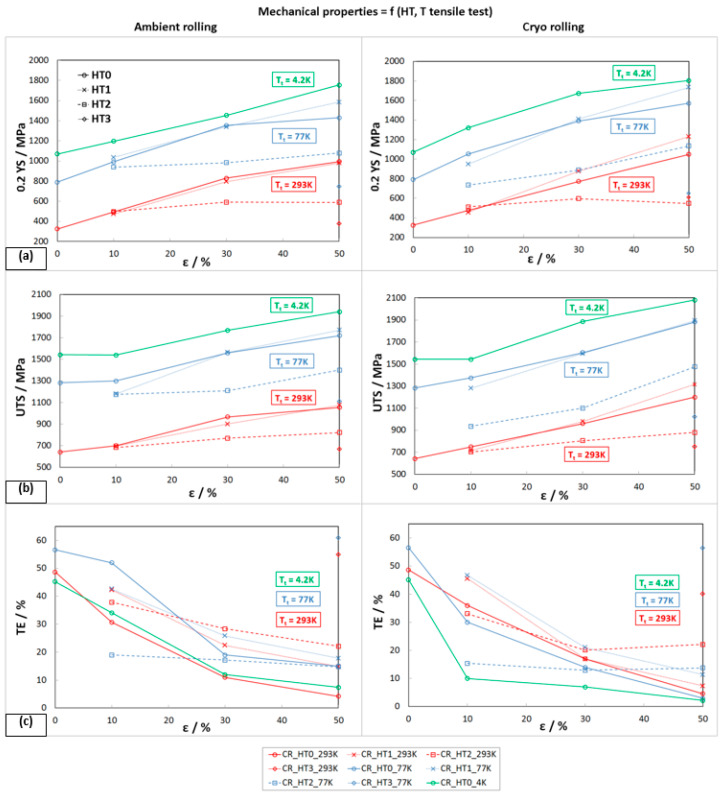
Comparison of mechanical properties for all HT procedures for AR and CR at three tensile test temperatures T_t_: (**a**) yield strength 0.2YS, (**b**) ultimate tensile strength UTS, (**c**) total elongation TE.

In Figure 13, the comparison of mechanical properties for all heat treatments after AR and CR for three tensile test temperatures is shown. Based on this comprehensive evaluation, it can be concluded that with increasing deformation (10, 30, and 50%), there is a significant strengthening of stainless steel 316LN-IG, while the strengthening is more intense when rolling in cryogenic conditions. It is best seen after 50% deformation (compare the solid red lines in Figure 13a,b). Furthermore, the strength properties are also strongly dependent on the testing temperature. The studied steel with a decrease in the testing temperature shows an increase not only in strength properties but, in some cases, also in plastic properties (compare the solid red and blue lines in Figure 13c, AR). The effect of heat treatment, according to the conditions of HT1, has a variable effect on the mechanical properties; in the case of 10% deformation, it reduces them, and in the case of 50% deformation, it increases them. These differences are probably due to the different precipitation hardening, since the strain-hardened structure affects the precipitation temperatures.

The annealing conditions of HT2 evidently reduce the strength properties (see dashed lines in Figure 13) compared to the deformed state. This reduction is caused by partial recrystallization of the structure.

#### 3.3.2. Hardness

**Figure 14:**
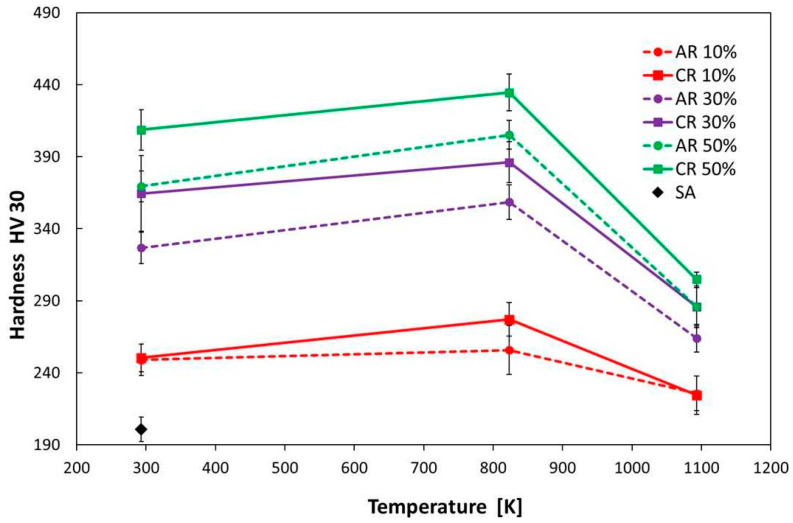
Vickers hardness measurement for samples of stainless steel rolled and annealed at different temperatures.

The changes in the hardness of stainless steel 316LN-IG as a function of annealing temperature (823 K and 1093 K) are shown in Figure 14. It can be seen from the dependence that, during annealing to a temperature of 823 K (HT1), there is an increase in hardness, on average by 30 HV. It can be stated that the hardness measurement is more sensitive than YS for monitoring the effect of hardening. This increase in hardness is due to precipitation hardening, which was also identified by calorimetric measurement. The highest hardness values were achieved after 50% cryo-rolling of 434 HV30. The significant decrease in hardness after annealing at 1093 K (HT2) corresponds to the thermal instability of the precipitates and the partial recovery of the deformed structure. In the case of 50% deformation, there was a drop of more than 120 HV30. A comparable development of hardness due to annealing was also presented in studies [6,39].

#### 3.3.3. The Structure Development after Plastic Deformation and Heat Treatment

**Figure 15:**
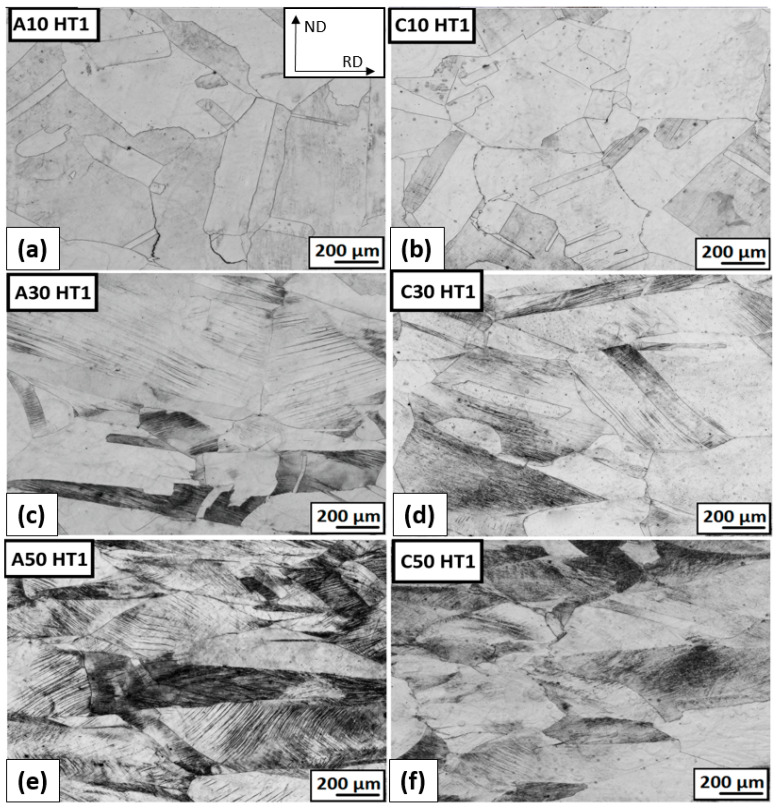
The microstructure overview after ambient and cryogenic rolling with different total-thickness deformations and heat treatment at 823 K for 10 h (HT1): (**a**) AR; ε = 10% (**b**) CR; ε = 10% (**c**) AR; ε = 30% (**d**) CR; ε = 30% (**e**) AR; ε = 50% (**f**) CR; ε = 50%.

The micrographs of the deformed structure after ambient and cryogenic rolling followed by heat treatment at 823 K for 10 h are shown in Figure 15. The structure after HT1 shows only minimal changes compared to the deformed state. In some cases, a subtle restoration of the structure can be observed. After ε = 10% cryo-rolling, the deformation bands are not observed. At higher degrees of deformation, high-deformation heterogeneity prevails. In the case of ε = 50%, the original grain edges are more visible after HT1. Due to long-term heat treatment, the precipitation process is expected, as well. The presence of precipitates cannot be confirmed due to the low resolution of the optical microscope.

**Figure 16:**
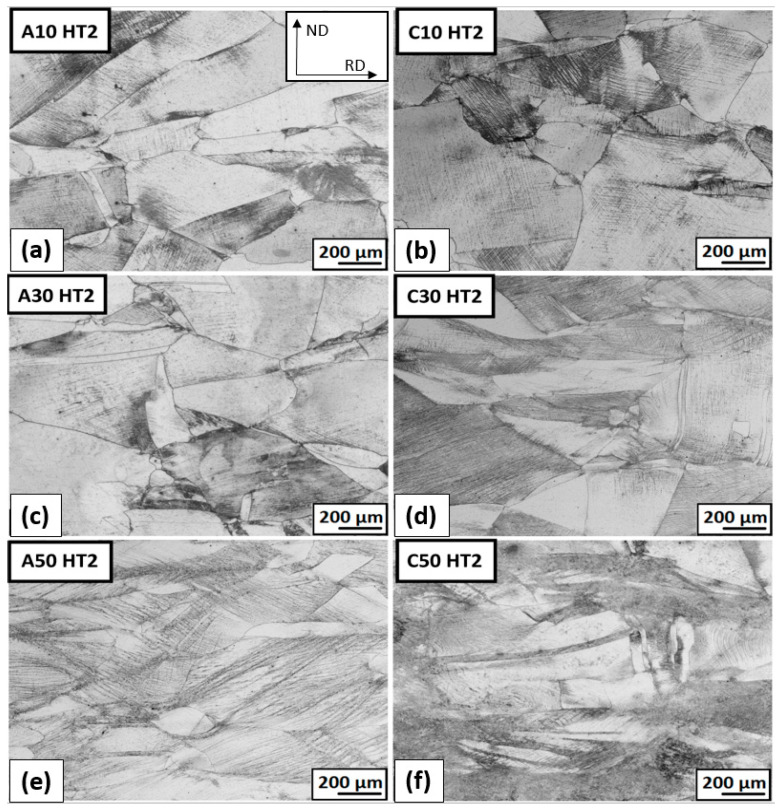
The microstructure overview after ambient and cryogenic rolling with different total-thickness deformations and heat treatment at 1093 K for 10 h (HT2): (**a**) AR; ε = 10% (**b**) CR; ε = 10% (**c**) AR; ε = 30% (**d**) CR; ε = 30% (**e**) AR; ε = 50% (**f**) CR; ε = 50%.

**Figure 17:**
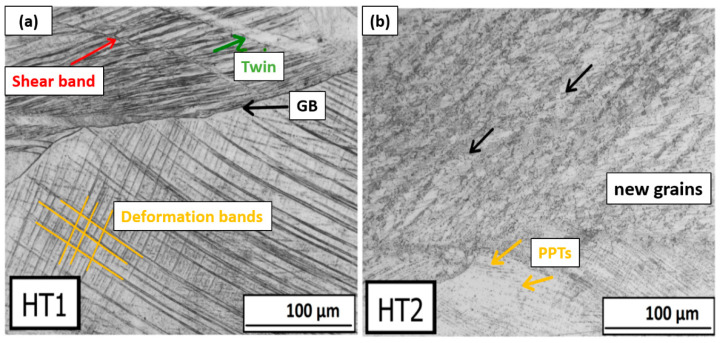
Comparison of selected areas of ε = 50% ambient rolling and two types of heat treatment: (**a**) HT1 (**b**) HT2 (PPTs—precipitates).

The micrographs of the deformed structure after ambient and cryogenic rolling followed by heat treatment at 1093 K for 10 h are shown in Figure 16. More advanced structure recovery is obvious. The grain boundaries of bigger grains elongated in a rolling direction are clearly visible. In areas with a high number of dislocations between bigger grains, small austenitic grains are observed. These areas are dark gray and black in the micrographs. A comparison of selected areas of total deformation ε = 50% of two types of heat treatment (HT1 and HT2) is shown in Figure 17. It can be said that after HT2, small grains of austenite are visible, while after HT1, shear bands are still present. This finding suggests that partial recrystallization may have occurred under HT2 conditions. After HT1, the criss-cross reaction of bands from the rolling process is obvious.

The grain size of ambient AS rolled by 30% and heat-treated AS at 823 K is 55 μm for ambient rolling and 9 μm for cryo-rolling. The difference between grain sizes may be caused by a partial recovery process or, in the case of cryogenically rolled material, by the new grains. These small grains reduce the average grain diameter rapidly. The material cryo-rolled by 30% and the heat-treated SA at 1093 K have an average grain diameter of approx. 10 μm for both types of the rolling process. The difference in microstructure is in the LAGB/HAGB ratio and the ratio of annealing twins. This will be examined through further analysis. The comparison of the microstructure of AR 50% after HT1 and HT2 is shown in Figure 17.

According to the lower temperature of HT1, partial recovery is obvious, but the material is stored at a high level of strain. After HT2, new austenite grains with an average diameter of 3–5 μm and precipitates are visible (Figure 17b).

**Figure 18:**
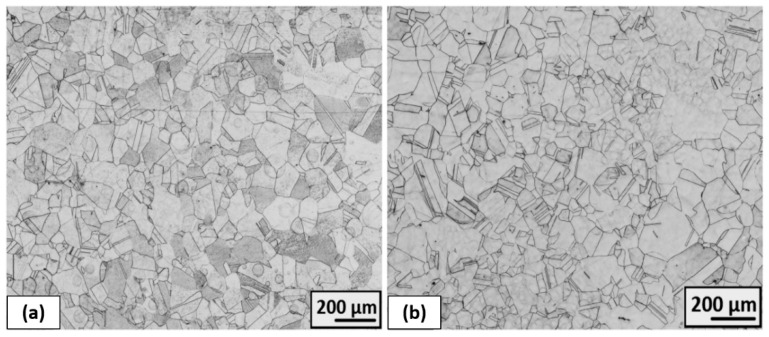
The microstructure overview after (**a**) ambient and (**b**) cryogenic rolling with 50% total- thickness deformation and heat treatment at 1323 K for 1.5 h (HT3) [30].

The completely recrystallized austenitic structure was observed after 50% deformation and following heat treatment at 1323 K for 1.5 h (Figure 18). Some annealing twins are visible, and all grains have clear boundaries. Grain size is characterized by the number G = 4.5, which corresponds to the value of the average grain diameter of 75.5 μm in the case of ambient rolling as well as cryogenic rolling.

## 4. Conclusions

Tensile properties of austenitic stainless steel 316 LN-IG were investigated in the temperature range 293–4.2 K under different conditions of heat-deformation treatment. Mechanical properties after rolling at ambient temperature and in liquid nitrogen, as well as after subsequent heat treatment, were compared. Individual stages of thermomechanical processing were analyzed using optical microscopy. The main results are summarized as follows:

- Austenitic stainless steel 316 LN-IG, after solid solution annealing, shows excellent synergy between strength and plastic properties at cryogenic temperatures. The strength of the steel increases continuously with decreasing testing temperatures (UTS_293K_ = 641 MPa, UTS_77K_ = 1282 MPa, and UTS_4.2K_ = 1543 MPa). Even at very low temperatures, the material shows high ductility TE_SA,77K_ = 57% and TE_SA,4.2K_ = 45%.
- The work-hardening rate of the steel increased continuously with a lowering of the temperature.
- After 50% deformation of thickness, a strongly deformed microstructure made up of tangles of deformation bands, shear bands, and twins was observed.
- The highest precipitation hardening of steel was achieved at a temperature of 823 K (HT1) after 50% deformation.
- At an annealing temperature of 1093 K, there was a loss of deformation and precipitation hardening in the steel.
- The completely recrystallized austenitic structure was observed after 50% deformation and following heat treatment at 1323 K for 1.5 h.

## Figures and Tables

**Table 1:** Heat treatment conditions for experimental rolling at ambient and cryogenic temperatures.

Heat Treatment	Temperature/K	Holding Time/h
SA	1323	1
HT0	as rolled	-
HT1	823	10
HT2	1093	10
HT3 (for A50 and C50)	1323	1.5

**Table 2:** Labeling of samples.

	Total Thickness Deformation ε/%					
Heat Treatment	10% Ambient	10% Cryo	30% Ambient	30% Cryo	50% Ambient	50% Cryo
HT0	A10 HT0	C10 HT0	A30 HT0	C30 HT0	A50 HT0	C50 HT0
HT1	A10 HT1	C10 HT1	A30 HT1	C30 HT1	A50 HT1	C50 HT1
HT2	A10 HT2	C10 HT2	A30 HT2	C30 HT2	A50 HT2	C50 HT2
HT3	-	-	-	-	A50 HT3	C50 HT3

## Data Availability

Not applicable.
